# Classification of human breathing sounds by the common vampire bat, *Desmodus rotundus*

**DOI:** 10.1186/1741-7007-4-18

**Published:** 2006-06-16

**Authors:** Udo Gröger, Lutz Wiegrebe

**Affiliations:** 1 Department Biologie II der Ludwig-Maximilians-Universität München, Großhadernerstr. 2, D-82152 Planegg-Martinsried, Germany

## Abstract

**Background:**

The common vampire bat *Desmodus rotundus *is one of three bat species that feed exclusively on the blood of mammals often more than 1000 times its size. Vampire bats even feed on human blood. Moreover, they tend to feed on the same individual over consecutive nights.

**Results:**

Using psychoacoustical methods, we show that vampire bats can recognize individual humans by their breathing sounds. Accompanying psychoacoustical experiments using the same stimuli and procedure but with human listeners show that even these trained and instructed listeners were unable to achieve the vampire bats' performance under the most difficult conditions, where the breathing sounds had been recorded under physical strain.

**Conclusion:**

It is suggested that vampire bats can make use of an individual acoustic signature imposed on breathing sounds in a way similar to that in which we identify humans by their vocalizations.

## Background

**Figure 1:**
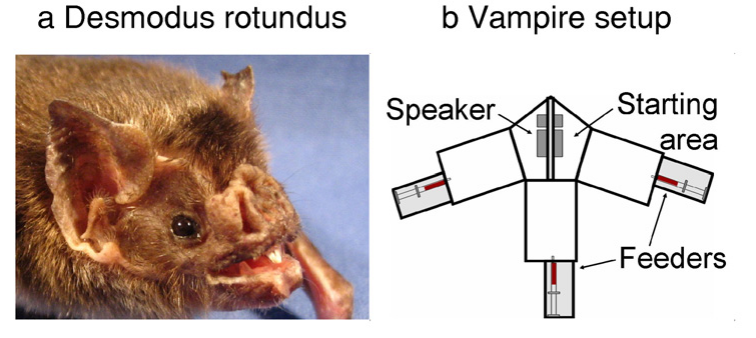
**Animal portrait (a) and experimental setup (b) for the behavioral experiments on breathing-sound classification and absolute thresholds in the common vampire bat, *Desmodus rotundus***. For the breathing-sound classification, animals were trained to associate each of three breathing sounds, presented from a speaker above the starting area, with one of the three feeders. For the absolute-threshold measurements, stimuli were presented from one of three speakers mounted above the feeders.

Vampire bats are the only mammals that feed exclusively on blood. The common vampire bat, *Desmodus rotundus*, is one of three vampire species (Fig. 1a). Typically, *D. rotundus *only feeds on a single prey animal on any one night. The blood meal lasts from 10 min up to 1 hour during which time the vampire bat drinks between 0.5 times and 1.4 times its body weight in blood. Despite the abundance of prey animals since the start of livestock farming, vampire bats repetitively feed on the same individual whereas other individuals are ignored [1-3]. The perceptual features that guide the vampire bats' selection of prey animals and enable them to recognize an animal that they have fed on the night before remain elusive.

Earlier studies revealed sensory specializations that support the extraordinary food and feeding strategies of *D. rotundus*: Kurten and Schmidt [4] found pit organs in the nose of *D. rotundus *that are sensitive to the infrared radiation emitted by the blood-rich skin surfaces of homeothermic vertebrates. However, the detection range for this infrared emission is only 8 to 12 cm [5]. Thus, infrared sensitivity cannot help to locate or select the prey animal but will identify a promising place to bite it. *D. rotundus *has a very well developed olfactory system. Both anatomical [6] and behavioral [7] studies indicate that olfaction may play an important role in both the long-distance orientation towards potential prey and possibly the selection of individual prey animals.

The importance of passive hearing, as opposed to echolocation, for the common vampire is supported by the very low thresholds of midbrain neurons in the frequency range between 10 and 25 kHz, i.e. considerably below the echolocation frequency range (~40 to 100 kHz). Moreover, these recordings have revealed neurons that are stimulated exclusively by breathing sounds [8].

**Figure 2:**
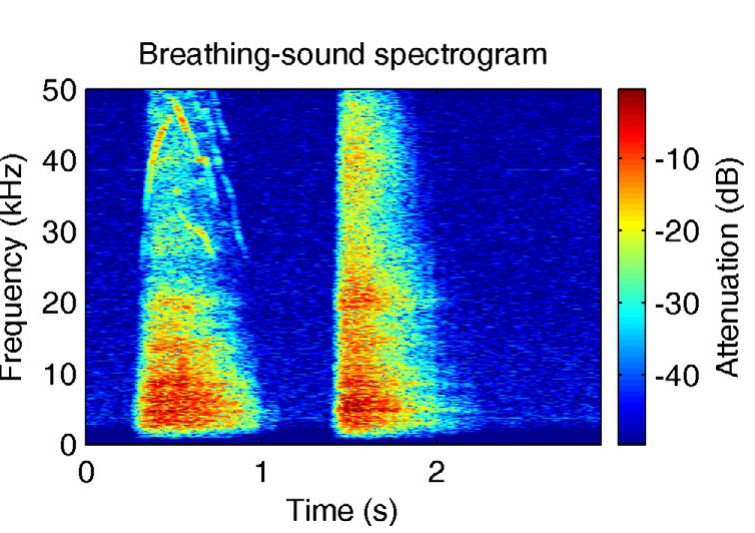
**Spectrogram of a recorded breathing sound**. The spectrogram shows the spectro-temporal features of an air intake (left) and output (right) in a single breathing-sound cycle. Frequency-modulated, ultrasonic tonal components can be observed during air intake. These components result from turbulences in the nasal cavity during depression. Note the good signal-to-noise ratio, which was realizable with the recording equipment described in the methods.

This study was designed to investigate the passive-hearing capabilities of the common vampire bat behaviorally; specifically, the extent to which auditory sensitivity to breathing sounds may support prey selection. Vampire bats were trained to discriminate three sequences of breathing sounds recorded from three different subjects. A spectrogram of a recorded breathing sound is shown in Fig. 2. Once the vampire bats had learned this task, additional breathing sounds, recorded under different experimental conditions from the same three subjects, were randomly interspersed. The spontaneous association of these test sounds with the learned training sounds was assessed. These psychophysical data from the vampire bats were compared to the performances of human listeners using the same experimental paradigm and stimuli. The experimental data, together with simulations of the breathing-sound task based on different sound parameters, allow the relevance of these parameters to the perceptual association of breathing sounds in both vampire bats and humans to be evaluated.

## Results

### Vampire behavior

**Figure 3:**
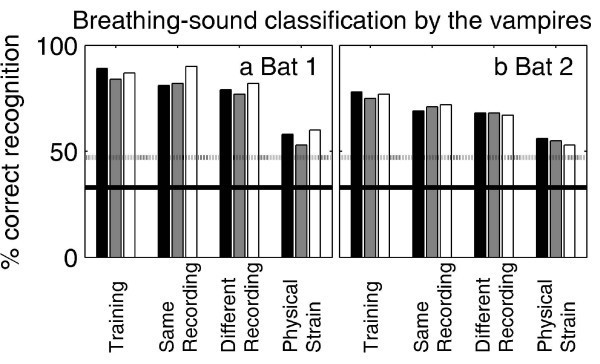
**Spontaneous classification of unknown breathing sounds by *D. rotundus***. The two panels show results from two individuals. The correct recognition of a breathing sound recorded from one of three human subjects is shown as a function of the condition of breathing-sound recording. The different bar colors represent the three different subjects. The bold horizontal line at 33 % correct indicates chance level, the gray line at 47 % correct indicates significant deviation from chance with p < 0.05. The data show that both animals recognized the breathing sounds correctly under all experimental conditions. Only under the most difficult condition, where the sounds were recorded under physical strain (unlike the training sounds), the bats' performance dropped markedly but still remained significant.

**Figure 4:**
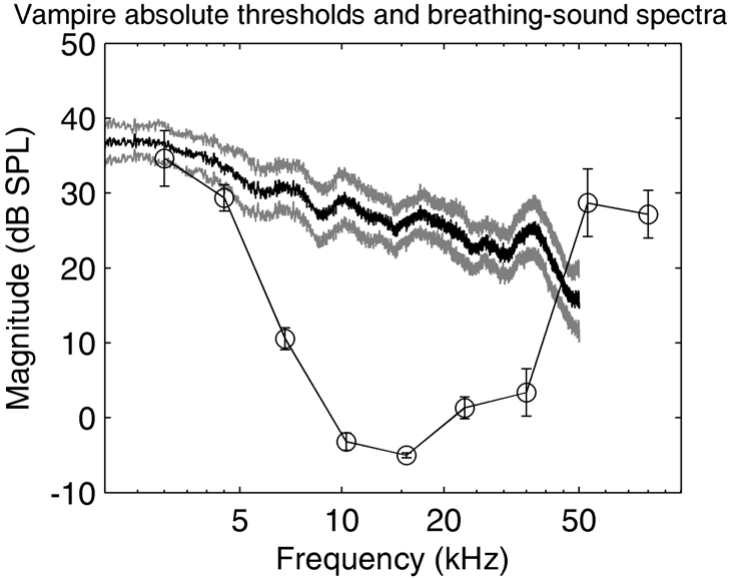
**Vampire hearing and breathing-sound spectra**. Absolute auditory thresholds averaged across three individuals of *D. rotundus *are shown as symbols and the fine line; error bars represent standard errors of the mean. Averaged breathing-sound magnitude spectra are shown by the strong black line; the strong grey lines again represent standard errors. This comparisons show that in *D. rotundus*, auditory sensitivity is well suited to detecting and analyzing breathing sounds.

The percentage spontaneous associations of nine unknown test sounds with the correct subject are shown in the two panels of Fig. 3 for the two vampire bats, respectively. The data show that the vampire bats reliably associated these test sounds with the correct subject (p < 0.05 [9], horizontal dotted lines). The vampire bats were even able to recognize the recordings under physical strain, although performance dropped markedly for this test-sound condition. The importance of passive hearing (as opposed to echolocation) is reflected in the very low auditory thresholds in the frequency range of breathing sounds. The absolute thresholds for long-duration narrow-band noise stimuli are shown as a function of the band-pass center frequency by the fine lines and circles in Fig. 4. Data are averaged across three vampire bats; error bars represent standard errors. The bold line in Fig. 4 shows the averaged breathing-sound spectra. The gray lines represent standard errors. These data show that the high auditory sensitivity of the vampire bat in the frequency range between 10 and 25 kHz is well suited to the detection and analysis of the breathing sounds we recorded. Owing to the noisy, broadband nature of breathing sounds, it is not to be expected that breathing sounds from other mammals of comparable size differ markedly from human breathing sounds in their magnitude spectra.

### Human behavior

**Figure 5:**
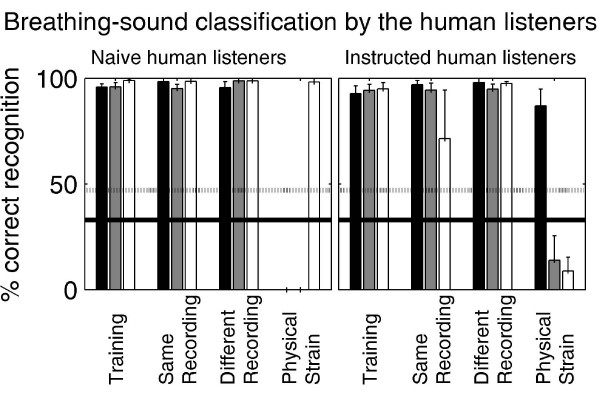
**Breathing-sound classification by human listeners with exactly the same stimuli and experimental procedure as used in the vampire experiment**. Results are shown in the same format as in Fig. 3. Error bars represent across-listeners standard errors. While the human listeners were well capable of recognizing breathing sounds recorded in the same session or in a different session in the same physical state, the naïve human listeners failed to recognize the breathing sounds recorded under physical strain (a). Even after the listeners were instructed to ignore breathing-frequency information (b), overall performance did not improve for this most difficult experimental condition.

Breathing-sound classification by human listeners is shown in Fig. 5 in the same format as Fig. 3. The human listeners were well capable of correctly associating the test sounds, recorded either in the same recording session or in another session under a similar degree of physical strain of the subjects. However, unlike the vampire bats, the naïve listeners (left panel) completely failed to classify the breathing sounds recorded under physical strain correctly. Instead, all sounds recorded under physical strain were consistently associated with subject #3. This subject used the highest breathing frequency in the training set. Breathing frequency is simply defined as the number of breathing cycles per second. Note that the human listeners went through the same training procedure as the vampire bats and scored at least as well as the vampire bats in the training condition and in the first two test conditions.

After completion of this data set, listeners were instructed to ignore breathing-frequency information but to try instead to exploit all other information. This was done because both the listeners' reports and the numerical simulations (see below) suggested that the listeners had been trying to use breathing-frequency information to classify the breathing sounds. This instruction had obviously not been given to the vampire bats. The results obtained under this experimental condition are shown in the right panel of Fig. 5. While the pattern of results changed in comparison to the naïve condition, listeners still failed to associate breathing sounds recorded under physical strain correctly.

### Simulations

**Figure 6:**
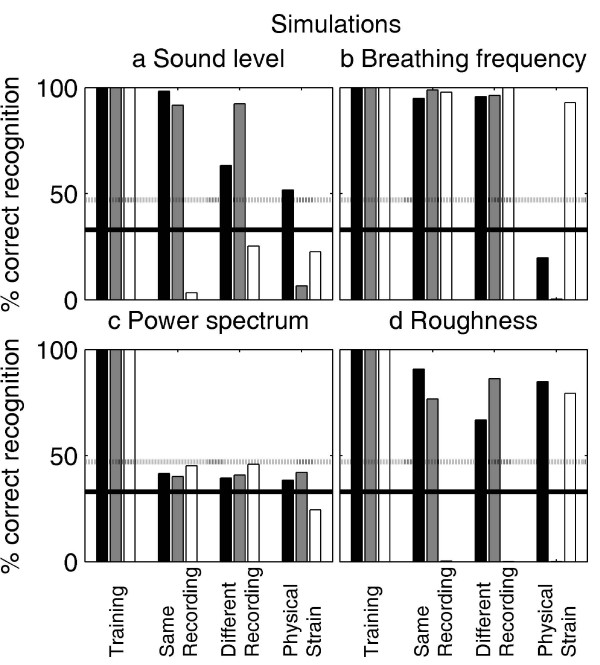
**Numerical simulation of the breathing-sound recognition**. The simulations are based on four different breathing-sound parameters, namely (a) the sound level, (b) the breathing frequency, (c) the power spectrum of the breathing sounds and (d) the breathing-sound roughness. None of the simulations matches the behavioral performance of the vampire bats (Fig. 3). The breathing-sound simulation, however, matches the performance of the naïve human listeners (Fig. 5a), confirming the listeners' reports that they had tried to rely on breathing-frequency information.

The numerical simulations of the breathing-sound association were implemented to assess whether the vampire bats' or humans' performances can be linked to a physical sound parameter. Simulation results for the four extracted sound parameters are shown in the four panels of Fig. 6. Overall, the simulation based on breathing frequency provides the best results while the power-spectrum simulation provides the worst. However, none of the simulations can correctly classify all test sounds recorded under physical strain. Note the high similarity between the breathing-frequency simulation (Fig. 6b) and the performance of the naïve human listeners (Fig. 5, left panel). This similarity supports the hypothesis that human listeners relied on breathing-frequency analysis.

These analyses were performed on the sounds as they were recorded. The audiogram of a vampire bat, however, is quite different from that of humans. To capture the possible effects of the different frequency ranges in which the bats and the humans processed the sounds, the simulations were repeated with the sounds filtered to match either the human or the vampire-bat audiogram. These simulations were only carried out for the three parameters that are potentially influenced by the filtering, namely sound-pressure level, differences in the magnitude spectrum and stimulus roughness. However, none of these parameters alone yielded significantly better predictions with the filtered sounds (not shown).

It is conceivable that the bats did not rely on a single sound parameter but on different parameters depending on which parameter yielded the strongest predictions for a given comparison of a test sound with the three training sounds. Although the magnitude spectrum did not provide strong predictions for a given test sound (the mean-squared difference between the magnitude spectrum of the test sound and each training sound was similar), the stimulus roughness provided strong predictions e.g. in favour of the training-sound of subject one.

**Figure 7:**
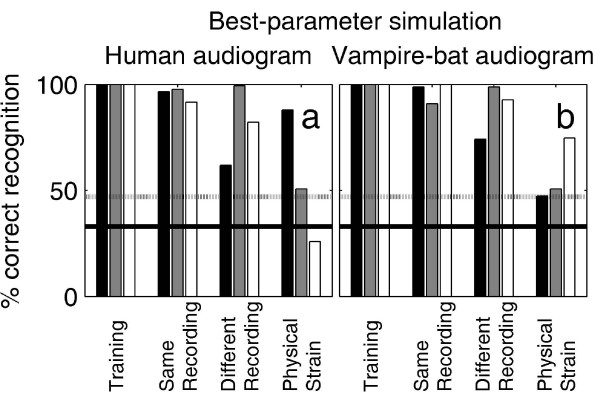
**Simulation of the breathing-sound recognition with dynamically changing analysis parameters**. Predicted breathing-sound classification when the model was free to choose the breathing-sound parameter (sound level, power spectrum or roughness) that provides the strongest predictions (strongest deviations from chance performance) for a given comparison of a test sound with each of the three training sounds. All sounds were filtered to match either the human audiogram (a) or the vampire-bat audiogram (b). While the simulation based on the human audiogram does not yield improved predictions of the classification of the test sounds recorded under physical strain, this simulation approach when combined with the sounds as they are weighted by the vampire-bat audiogram results in qualitatively correct classifications even of the test sounds recorded under physical strain.

Consequently, a simulation was implemented in which the model was free to choose the parameter for a given test sound that provided the strongest predictions. Again, test sounds were either filtered to match the human or the vampire-bat audiogram. Simulation results are shown in Fig. 7. While the predictions of this model are still poor when the sounds were filtered to match the human audiogram (Fig. 7a), they were quite good in predicting the bats' performance when the sounds were filtered to match the vampire-bat audiogram.

In summary, the current functional simulations suggest that the vampire bats spontaneously recruited a rather sophisticated analysis of the sounds based on multiple parameters; and they appeared to base decisions for each test sound on the sound parameter that provided the strongest discriminative capacity for comparing this test sound with the three training sounds. It appears that this analysis is more reliable when based on ultrasonic components of the sounds.

## Discussion

The current data show that for vampire bats, prey-generated breathing sounds could provide a reliable cue for recognizing prey individuals: during the relatively long time a vampire feeds on a prey animal, it can memorize the prey's breathing sounds and use this information to find the same prey on the following night.

Breathing sounds are typically faint. The sounds we recorded from human subjects ranged between 25 and 35 dB SPL. This gives rise to the question: over what distance could breathing sounds be perceived and analyzed by vampire bats? Considering the absolute thresholds (cf. Fig. 4), the frequency region most likely to be used is around 15 kHz, where thresholds are as low as 0 dB SPL. In this frequency region, atmospheric attenuation is around 0.5 dB/m. Thus, in the (unlikely) absence of any masking sounds, detection of breathing sounds could work over several tens of meters. In the presence of natural masking sounds, however, the effective detection distance will depend on the level and spatial distribution of the masking sound sources.

While it is unlikely that prey recognition relies exclusively on breathing sounds [5,7], these sounds potentially have high individual significance: vocalizations are generated by the vocal cords and filtered through the vocal tract. Both the pattern of vocal-cord vibrations and the filtering are highly individual and this supports our recognition of individual voices. While breathing sounds are unvoiced, and thus do not excite the vocal cords, they will also pass the same vocal and nasal tract and may thus also mediate individually specific information. However, the sounds used in this study were emitted through the nose. It remains to be investigated to what extent the nasal acoustic tract filters breathing sounds in a similarly characteristic way. An early study confirmed that, at least for partially voiced sounds such as consonant-vowel combinations, speaker recognition is feasible on the basis of nasal co-articulation [10]. However, the current simulations based on the breathing-sound power spectra, and the human-psychophysical experiments, suggest that speaker recognition is difficult with purely unvoiced sounds. If nasal-tract filtering were individually specific, the resulting spectral features would result in a correct breathing-sound association in the power-spectrum simulation. The simulation results in Fig. 6c show that this is not the case. Also, the failure of the instructed human listeners to associate the breathing sounds recorded under physical strain argues against the use of power-spectrum information for breathing-sound recognition, at least in the audio frequency range below 20 kHz. Note that human listeners are very sensitive to changes in the spectral composition of broadband stimuli [11].

Qualitatively correct predictions of the vampire-bats' performance, even under the most difficult experimental condition where the test sounds had been recorded under physical strain, could be obtained with a refined simulation approach: first, the sounds were filtered to match the vampire-bat audiogram; and second, a simulation paradigm was designed that allows whichever sound parameter yields the strongest predictions to be exploited.

## Conclusion

The current behavioral study shows that the common vampire bat, *Desmodus rotundus*, is very sensitive to breathing sounds. In the three-alternative, forced-choice setup, it spontaneously associates unknown breathing sounds with the subject who emitted them. This exceptional performance is underlined by the inability of human listeners to match the vampire bats' accomplishment under the most difficult experimental condition where the sounds had been recorded under physical strain. Numerical simulations show that while the human listeners relied on breathing-frequency information, the vampire bats appeared to recruit different acoustic parameters and to choose amongst these parameters depending on which provided the highest discriminative power.

On the basis of these findings, it is suggested that vampire bats can memorize and classify complex acoustic features of prey-generated breathing sounds to facilitate the identification of prey animals that they have successfully fed on before.

## Methods

### Breathing-sound recordings

Breathing sounds were recorded from three human subjects (two female, one male) aged between 27 and 30 and breathing through the nose. The microphone (Bruel&Kjaer 4189 with B&K 2671 preamplifier, Naerum, Denmark) was positioned at a horizontal distance of 10 cm and 2 cm below the nose tip. This position was found after extensive tests to ensure maximum acoustic sensitivity to the breathing sound while, at the same time, minimizing the risk of the air stream hitting the microphone directly. Care was taken to exclude recordings where the air stream hit the microphone. The microphone was connected to a B&K 2525 measuring amplifier; the output was high-pass filtered at 1 kHz (Krohn Hite 3550, Brockton, MA) and analog-digital converted (Tucker Davis Technologies RP2.1, Alachua, FL) at a sampling rate of 100 kHz. This recording system had a flat frequency response up to 35 kHz followed by a shallow decay (about 12 dB/oct.), caused by the 1/2 inch microphone. This microphone was chosen because, unlike a ¼ inch microphone, the background level was low enough to obtain a reasonable signal-to-noise ratio of 20–25 dB with the breathing sounds. Three 40-s sessions were recorded from each subject. One session was recorded after the subject was subjected to physical strain (20 knee bends).

The breathing sounds used to train the vampire bats consisted of a sequence of full breathing cycles lasting between 7 and 10 s extracted from the first recording session. The test sounds also consisted of full cycles lasting between 7 and 10 s extracted (1) from a different time period within the same 40-s session, (2) from a second 40-s session recorded on a different day and (3) from the session recorded under physical strain. Thus, the test program consisted of nine test sounds (three subjects times three test conditions). Downsampled (to 44.1 kHz) and compressed versions (mp3, 128 kB/s) of all training and test sounds are provided in the supplementary information.

### Stimulus presentation

The breathing sounds were digital-analog converted at 100 kHz (TDT RP2.1), amplified (Rotel RB 976 MkII, Worthing, England) and presented through a speaker (Technics Matsushita EAS10TH800D, Osaka, Japan) mounted vertically above the starting area of the vampire. Emission sound levels were set to the recorded levels (25 to 35 dB SPL) including their natural intra- and inter-individual variability. The frequency response of the playback system was flat (± 2 dB) between 3 and 48 kHz.

### Procedure

In a three-alternative, forced-choice paradigm, the vampire bats (Fig. 1a) were trained to associate each of the three training sounds with a corresponding reward feeder located at the ends of the three arms of the behavioral setup (Fig. 1b). When the vampire arrived at the correct reward feeder before the end of the stimulus presentation, it was rewarded with 0.25 ml of cattle blood provided by an automated syringe system under computer control. A video clip of a vampire approaching a feeder and feeding is provided in the supplementary information [see Additional files 2 and 3]. Two of the four animals trained on this task learned to associate each training sound with a specific feeder with more than 70 % correct performance after about 6 months of training. Test trials were then randomly interspersed between the training trials with a probability of 25%. In these test trials, one of the nine test sounds was presented and the vampire bats were rewarded independently of their choice of reward feeder. Whether a trial was a training trial or a test trial, and which feeder would be the correct feeder for the training trials, were determined exclusively by random generators in the software. Control of the automated syringes was done over the IO port of the TDT RP2.1 and this switching only occurred after the animals had made a decision. The results shown are based on at least 30 presentations of each of the nine test sounds. Thus, the data result from at least 270 test trials interspersed between 810 training-sound presentations. Numerical simulation based on random performance shows that the p < 0.05 threshold in this 3-AFC task with 30 trials per condition is 47% correct [9]. The data-acquisition period lasted about one year.

### Absolute threshold measurements

#### Stimuli

Absolute auditory thresholds were determined for nine center frequencies between 3 and 80 kHz equally spaced on a logarithmic frequency axis. The stimuli were narrow-band noises with a -3 dB bandwidth of the center frequency ± 10 %; the noises were regenerated for each trial. Each noise had a duration of 500 ms including 10 ms raised-cosine ramps. Stimuli were presented through the TDT RP2.1, a TDT PA5 programmable attenuator, the Rotel RB 976 MkII, and a 40 dB passive end attenuation. The end attenuator consisted of a 100 Ω resistor in series and a 1 Ω resistor in parallel to each speaker. The speakers were Technics Matsushita (EAS10TH800D) and they were mounted at the ends of the three arms. The setup was calibrated with a ¼ inch microphone (B&K 4135) connected to a B&K 2670 preamplifier and a B&K 2636 measuring amplifier. Stimuli were presented at a rate of 1 Hz for 15 s or until the vampire had reached one of the feeders. Correct choices were rewarded in the same way as in the breathing-sounds experiment.

#### Procedure

Psychometric functions were obtained over an attenuation range of 35 dB in steps of 5 dB. The overall position of this attenuation range was set according to the individual animal's performance in preliminary trials. Within this 35-dB range, the attenuation for each trial was selected randomly. Each point on the psychometric functions is based on at least 30 trials; the 47 % correct point (corresponding to p < 0.05 in the three-alternative, forced-choice task) on a sigmoidal function fitted to the psychometric function was taken as threshold.

The shape of the psychometric functions, the frequency dependence of the absolute thresholds and the different associations of the test sounds are incompatible with the hypothesis that the animals relied on other than auditory cues to perform the task. In this context all trials in the absolute-threshold measurements with high attenuations, where the animals' performance was at chance level, can be regarded as blank trials. As the animals typically responded to 20 to 30 trials per day, the data for a single psychometric function required about 8 to 12 training days.

### Animals

Four adult *D. rotundus *were trained on the breathing sounds. Two animals failed to learn the association of the breathing sounds with the corresponding feeders and were removed from the experiment after 6 months of training. Three animals, two of them identical to those having taken part in the breathing-sound experiment, were subsequently trained on the absolute-threshold measurements. The animals were born in captivity in a colony located at the Zoologisches Institut, Universität Bonn, and they were kindly provided by Prof. Schmidt.

### Human psychophysical experiments

The breathing-sound experiment was repeated with four human listeners using exactly the same experimental stimuli and paradigm. The listeners were two males and two females (22 to 24 years old) and had normal hearing as determined by preceding measurements of absolute thresholds. The breathing sounds were delivered diotically through AKG K240DF headphones (Wien, Austria) at the recorded sound level. Instead of a blood reward, the listeners received visual feedback from a graphical user interface shown on a touch screen in a sound-proof booth. The touch screen also served as a response interface.

### Simulations

The simulations were performed to investigate whether the vampire bats' or the humans' performances could be linked to a simple physical sound parameter. Four such parameters (the sound-pressure level, the base-ten logarithm of the breathing frequency, the power spectrum and the breathing-sound roughness) were extracted from the three training sounds and the nine test sounds. The breathing-sound roughness was calculated as the 4^th ^moment of the waveform [12]. The fourth moment is the waveform raised to the power of four divided by the squared waveform raised to the power of two. As the divisor corresponds to the squared variance of the waveform, the division makes the 4^th ^moment independent of the sound-pressure level. For each of the four extracted breathing-sound parameters, the similarity between each training sound and each test sound was calculated as the reciprocal of the squared difference between the test sound and a training sound. For the power spectrum, the squared difference was averaged over all frequencies (0 to 50 kHz). Percentage correct identification was calculated by dividing the similarity between the test sound and the correct training sound (from the correct subject) by the sum of the similarities between the test sound and the training sounds from all three subjects.

To test the influence of the different hearing ranges of vampire bats and humans, the sounds were subsequently filtered with infinite-impulse response filters designed either to match the vampire audiogram (cf. Fig. 4) or the human audiogram [13]. Then the simulations for the three parameters that are potentially affected by this filtering (sound-pressure level, power spectrum and roughness) were repeated. However, the predictions of the breathing-sound classification by the vampire bats did not improve (not shown).

These simulations only evaluate the contribution of each the extracted parameters but not combinations of parameters. In the first simulations, the breathing-frequency parameter was identified as a distractor for the correct classification of breathing sounds recorded under physical strain. It is conceivable that while none of the other parameters alone can predict the vampire bats' performance, a combination of the remaining parameters, namely sound-pressure level, power spectrum and roughness, may be used by the vampire bats to classify breathing sounds.

To test this hypothesis, the model was allowed to choose the parameter that produced the strongest predictions, i.e. the strongest deviations from chance level, for the comparison of a given test stimulus with each of the training stimuli. Again, this version of the model was evaluated for both the human and the vampire hearing ranges. Simulation results are shown in Fig. 7. While the best-parameter simulation was still unable to achieve a significantly correct performance under the 'physical strain' condition when the sounds were filtered with the human audiogram, filtering with the vampire-bat audiogram resulted in qualitatively correct predictions even for those breathing sounds that had been recorded under physical strain.

## Authors' contributions

UG and LW designed the vampire experiments. UG carried out the animal training and data acquisition. LW implemented and carried out the human psychophysical experiments and simulations. UG and LW wrote the manuscript and designed the figures.

## Supplementary Material

Additional File 2 Videoclip of a vampire bat approaching an automated feeder Click here for file

Additional File 3 XviD is an ISO MPEG-4 compliant video codec, designed to compress/decompress digital video. This needs to be installed to view the .avi. Click here for file

Additional File 1 Sound files (mp3 format, 44.1 kHz, 128 kB/s) of all sounds used in the breathing-sound experiments. Click here for file
